# Lacerate and Macerate: The BASILICA–LLAMACORN/UNICORN Combination to Optimize Bioprosthetic Bileaflet Modification

**DOI:** 10.1016/j.jscai.2025.102614

**Published:** 2025-03-11

**Authors:** Jonathan X. Fang, Gennaro Giustino, Tiberio M. Frisoli, James C. Lee, Pedro A. Villablanca

**Affiliations:** a Center for Structural Heart Disease, Henry Ford Health System, Detroit, Michigan; b National Heart Centre Singapore, Singapore; c Gagnon Cardiovascular Institute, Morristown Medical Center, Atlantic Health System, Morristown, New Jersey

**Keywords:** BASILICA, coronary obstruction, electrosurgery, LLAMACORN, transcatheter aortic valve replacement, UNICORN

Bioprosthetic or native aortic scallop intentional laceration to prevent iatrogenic coronary artery obstruction during transcatheter aortic valve replacement (BASILICA)^1^ is the standard method of leaflet modification to prevent coronary obstruction. BASILICA typically requires 2 guides per leaflet, 1 for traversal and 1 for snaring. Concern of coronary obstruction in both leaflets is not uncommon. Bileaflet BASILICA could be a lengthy procedure. Lack of guide support and limitation in fluoroscopy angles may hinder right cusp BASILICA, resulting in the need to convert to chimney stenting or coronary obstruction due to incomplete leaflet splay following an insufficiently basal traversal.

**Figure 1:**
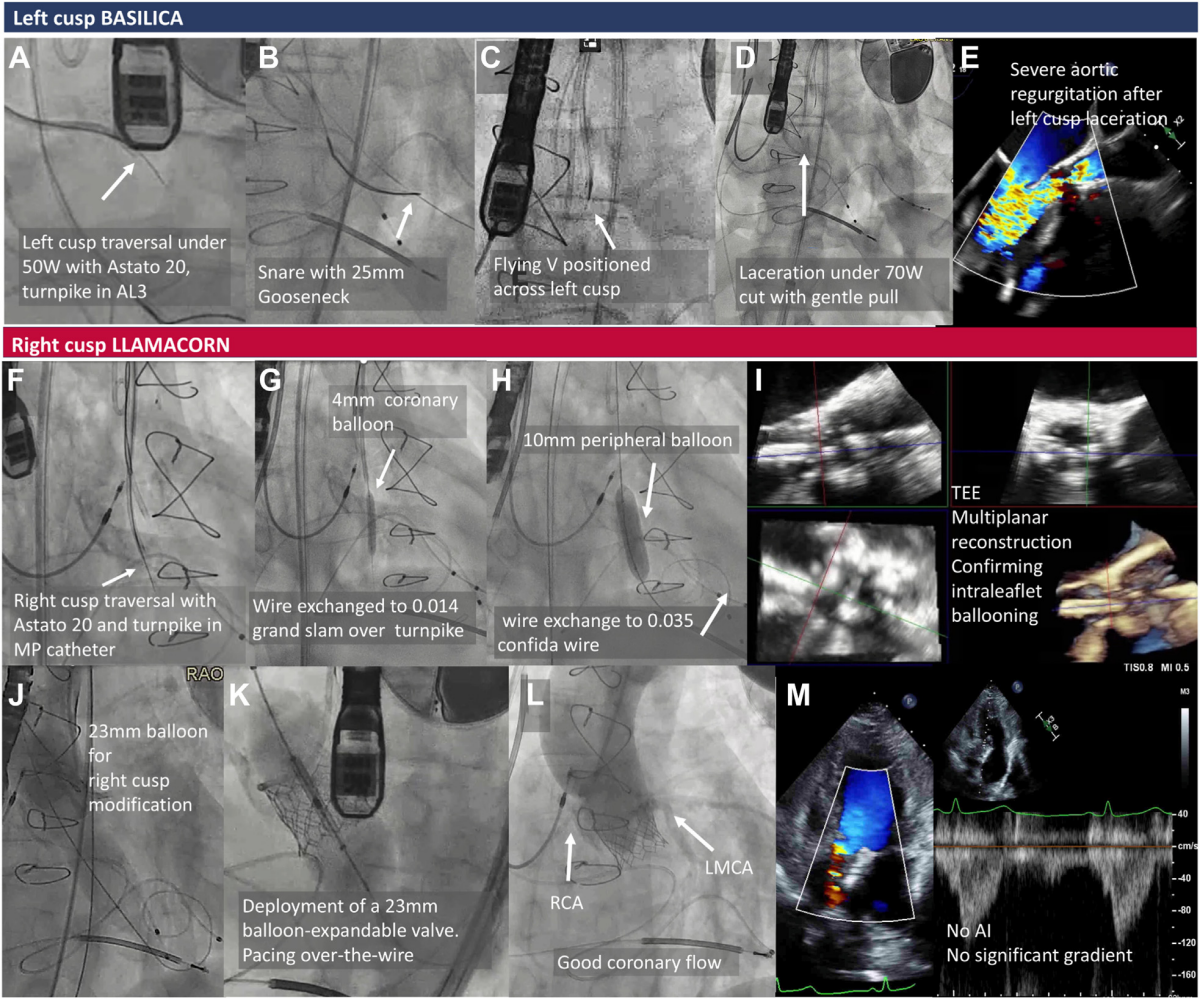
T**he BASILICA-LLAMACORN hybrid procedure for bileaflet modification.** (**A-E**) Left cusp bioprosthetic or native aortic scallop intentional laceration to prevent iatrogenic coronary artery obstruction during transcatheter aortic valve replacement (BASILICA). (**F-K**) Right cusp leaflet laceration with balloon mediated annihilation to prevent coronary obstruction with radiofrequency needle (LLAMACORN). (**L, M**) Results. AI, aortic insufficiency; LMCA, left main coronary artery; RCA, right coronary artery; TEE, transesophageal echocardiogram.

A 60-year-old man with a degenerated stentless surgical bioprosthetic valve required bileaflet modification for transcatheter aortic valve replacement owing to short valve-to-coronary distances. We performed left cusp BASILICA with Astato 20 wire (ASAHI INTECC) and Turnpike LP microcatheter (Teleflex) in an AL3 guide, snared by a 25-mm gooseneck (Figure 1A, B) and cusp laceration with a flying V under 70-W electrosurgery cut (Figure 1C-E). Owing to a challenging traversal angle and concern of insufficient splay for the right cusp, we performed a balloon-based leaflet modification instead of right cusp BASILICA. After traversal with Astato and Turnpike in a multipurpose guide (Figure 1F), we switched to a 0.014-inch Grand Slam wire (ASAHI INTECC) and created a track with 4-mm and 10-mm balloons with transesophageal echocardiogram confirmation of traversal position (Figure 1G-I). Then, we lacerated the leaflet with a 23-mm balloon and deployed a 23-mm balloon-expandable valve pacing over a Confida wire (Medtronic) exchanged through the 10-mm balloon (Figure 1J, K), achieving good result (Figure 1L, M).

Balloon augmentation can increase leaflet splay in BASILICA.^2^ Despite limited data, balloon-based leaflet modification is increasingly being performed.^3^ The term undermining iatrogenic coronary obstruction with radiofrequency needle (UNICORN) has been loosely applied to a variety of intraleaflet valve deployement or balloon-based leaflet modification techniques. The original procedure by Chan et al^4^ used a preformed 0.035-inch radiofrequency wire with a J tip (VersaCross; Baylis) for traversal and creation of a 10-mm tract on the leaflet with balloons without leaflet laceration, followed by intraleaflet deployement of a balloon-expandable valve. It has application in cases with extreme risk of coronary obstruction and enables leaflet modification without the use of snares or flying Vs. Despite its safety, the J-tip 0.035-inch wire is, in our experience, less effective than 0.014-inch wires in traversing calcified leaflets. Originally limited to balloon-expandable valves and single leaflet modification only, various modifications of the UNICORN procedure have been made to enable the use of self-expanding valves. An example is the leaflet laceration with balloon mediated annihilation to prevent coronary obstruction with radiofrequency needle (LLAMACORN) procedure^5^, which uses a 0.014-inch coronary wire for traversal and leaflet modification with a balloon sized to the aortic annulus prior to valve deployement to enable the use of self-expanding valves. It matches the technique used for the right cusp in our procedure. A BASILICA–LLAMACORN or UNICORN hybrid approach is potentially appealing for bileaflet modification, as it can reduce the number of access and overlapping guides required, allow more leniency on the precision required for right cusp traversal, and obviate the need of wire-snaring and the use of a flying V twice, although the serial balloon dilatation required could still be cumbersome. An important limitation of balloon laceration is the lack of directional control. It should be used with caution in calcified leaflets and bioprosthesis with long leaflets to avoid the creation of long scallops, which could avulse and cause coronary obstruction. Balloon laceration may also create more aortic regurgitation compared to BASILICA although the time required from laceration to valve deployement is shorter. Careful case selection and performance at high-volume centers is advised to ensure procedural safety.
